# Seasonality of mood and affect in a large general population sample

**DOI:** 10.1371/journal.pone.0239033

**Published:** 2020-09-14

**Authors:** Wim H. Winthorst, Elisabeth H. Bos, Annelieke M. Roest, Peter de Jonge

**Affiliations:** 1 Interdisciplinary Center for Psychopathology and Emotion regulation, Department of Psychiatry, University of Groningen, University Medical Center Groningen, Groningen, The Netherlands; 2 Department of Developmental Psychology, University of Groningen, Groningen, The Netherlands; King's College London, UNITED KINGDOM

## Abstract

Mood and behaviour are thought to be under considerable influence of the seasons, but evidence is not unequivocal. The purpose of this study was to investigate whether mood and affect are related to the seasons, and what is the role of neuroticism in this association. In a national internet-based crowdsourcing project in the Dutch general population, individuals were invited to assess themselves on several domains of mental health. ANCOVA was used to test for differences between the seasons in mean scores on the Positive and Negative Affect Schedule (PANAS) and Quick Inventory of Depressive Symptomatology (QIDS). Within-subject seasonal differences were tested as well, in a subgroup that completed the PANAS twice. The role of neuroticism as a potential moderator of seasonality was examined. Participants (n = 5,282) scored significantly higher on positive affect (PANAS) and lower on depressive symptoms (QIDS) in spring compared to summer, autumn and winter. They also scored significantly lower on negative affect in spring compared to autumn. Effect sizes were small or very small. Neuroticism moderated the effect of the seasons, with only participants higher on neuroticism showing seasonality. There was no within-subject seasonal effect for participants who completed the questionnaires twice (n = 503), nor was neuroticism a significant moderator of this within-subjects effect. The findings of this study in a general population sample participating in an online crowdsourcing study do not support the widespread belief that seasons influence mood to a great extent. For, as far as the seasons did influence mood, this only applied to highly neurotic participants and not to low-neurotic participants. The underlying mechanism of cognitive attribution may explain the perceived relation between seasonality and neuroticism.

## Introduction

According to the World Health Organization (1946) health is a state of complete physical, mental and social well-being (i.e. positive states) and not merely the absence of disease and infirmity (i.e. negative states) [1]. Mental problems cause considerable health loss and have a substantial economic impact on health service use and loss of productive capacity [2, 3]. Thus, the suffering caused by mental problems, the accompanying disability and the socio-economic consequences justify research on factors that influence the mental well- and ill-being of humans. Seasonality presumably is one of these factors.

The physical and mental state of humans is thought to be under the considerable influence of the seasons [4]. In the somatic domain, infectious diseases like influenza and allergic conditions like hay fever show a typical seasonal pattern. In the mental domain, the seasonal changes are considered to have an impact on human mood and behaviour as well, even leading to complaints and psychiatric illnesses like depression [5]. However, there is still an on-going debate to what extent the seasons influence the mental well- and ill-being of humans [6–14]. Studies using symptom questionnaires in the general population or specific questionnaires in selected populations suggest that the seasons have considerable influence on the mental state of individuals [15–21]. Other studies in the general population have failed to demonstrate a seasonal pattern or have shown only minimal seasonal differences in the prevalence rates of mental disorders [14, 22–24].

In the Netherlands, one study showed that 3% of the population suffers seasonally from serious depressive complaints described as Seasonal Affective Disorder (SAD), and 8.5% from mild depressive complaints [16]. In contrast to this finding, in a cohort of Dutch primary and secondary care patients with a major depressive disorder and anxiety disorders, seasonal differences in depressive and anxiety symptoms, as measured with a general screening instrument and symptom questionnaires, were absent or small [25]. In this same cohort, seasonal variation in mood and behaviour was reported by healthy controls and patients with a lifetime diagnosis of depression with or without a comorbid anxiety disorder [26]. Patients with more severe psychopathology more frequently reported seasonality of symptoms. Another study using the same cohort of patients showed that the seasonal distribution of major depressive episodes was not different for participants with or without SAD [27]. In the latter study, participants in the SAD group scored high on psychopathology and neuroticism throughout the year, but no differences were found for extraversion, openness, agreeableness and conscientiousness. Our studies led to the hypothesis that individuals who have high levels of psychopathology and who score high on neuroticism may attribute their unwell-being to a greater extent to the seasons than individuals with less severe complaints. Thus, neuroticism might be a moderator of the seasonal differences in (perceived) symptom severity.

Most studies focus on the seasonal recurrence of depressive episodes by measuring complaints and negative affect (NA) and not strengths and measures of well-being like positive affect (PA). However, contextual factors, positive affect and mental strengths are factors that can protect against mental problems and are equally worthwhile to adopt in research focusing on mental problems [28–30]. For this reason, we want to examine whether the seasons are equally related to positive and negative affect.

Another problem in most studies is the absence of repeated measures; as most studies on seasonality are cross-sectional. In the present study, we will use both cross-sectional data and repeated measures, to be able to examine seasonal within-subject differences as well. Finally, we will examine whether the personality factor neuroticism has an impact on seasonality.

We formulated the following research questions: 1) Are there any seasonal differences in the domains of positive affect, negative affect and depressive symptoms on a population level? 2) Can a within-person pattern of seasonality be shown in the domains of positive affect and negative affect in the subgroup of participants who filled out the questionnaires twice? 3) Is neuroticism a moderator of the relation between the seasons and positive and negative affect?

## Materials and methods

### Study design and participants

This study was conducted using data from the national internet-based crowdsourcing study HowNutsAreTheDutch (HND) (Dutch: HoeGekIsNL), which was designed to investigate the associations and dynamic interactions between mental strengths and vulnerabilities, both between and within participants, in a sample from the Dutch general population. A detailed description of the study can be found in Van der Krieke et al. [31].

In this project, individuals were invited to visit the website www.hownutsarethedutch.com and to assess themselves on several domains of mental health. In order to reach as many Dutch people as possible for inclusion in HND, publicity for HND was sought using several newspaper and magazine articles, public lectures, radio interviews, and other media.

Participants had to register, create an account with a password and submit their email address. They received an email with a hyperlink to confirm their registration. Participants started with an online inclusion procedure. Inclusion criteria were adulthood (18+) and informed consent on the use of their data for scientific research. Participants had to confirm this statement by checking the appropriate check box. After the inclusion procedure, they were given access to a module to assess date of birth, gender, postal code area and country of residence (the Netherlands, Belgium, other). After completion of this module, three key modules became available on living situation, affect/mood and well-being respectively. After this, all other modules became available. Completion of the following modules was optional, with not all participants completing all modules. All items of the self-report questionnaires had to be completed, and since blank items were not allowed, missing data were minimal. There were no formal exclusion criteria, but the HND study was presented in Dutch, which implied that citizens who could not read Dutch were unable to fill out the questionnaires unless they used a translator.

In order to examine possible selection effects, the sample of participants in the HND study was compared by Van der Krieke et al. [31] to the governmental data of the general Dutch population (Central Bureau of Statistics) and two large population studies: the Netherlands Mental Health Survey (NEMESIS-2) with 6,646 respondents and the Lifelines population study with 167,729 respondents [31–33].

The present study concerns the 14,489 individuals who participated between December 19^th^, 2013 (launching date of the internet platform) and December 19^th^, 2017 (fourth-year data extraction).

### Ethics statement

The Medical Ethical Committee of the University Medical Center Groningen approved the study procedures and declared the study was exempted from review by the Medical Research Involving Human Subjects Act (in Dutch: WMO) because it was a non-randomized open study targeting anonymous volunteers in the general population (registration number M13.147422). Inclusion criteria were adulthood (18+) and informed consent on the use of their data for scientific research. Participants had to confirm this by checking the appropriate checkbox at the online inclusion procedure. In the course of the study, it became apparent that after the introduction of the General Data Protection Regulation of the European Parliament (Regulation EU 2016/679), our dataset is considered pseudo-anonymized rather than anonymized, and is still regarded as personal data. Given that participants have not given informed consent to have their data publicly shared, legal and ethical considerations prevent public disclosure of our dataset. Data are therefore available upon request to the HowNutsAreTheDutch data access committee (info@hoegekis.nl).

### Modules and questionnaires

Participants were invited to complete various questionnaire “modules” covering specific psychological domains. The mood and affect module included the Positive and Negative Affect Schedule (PANAS), and the Quick Inventory of Depressive Symptomatology (QIDS). The personality module included the NEO Five-Factor Inventory-3 (NEO-FFI-3).

The present study concerns the 14,489 individuals who participated between December 19^th^, 2013 (launching date of the internet platform) and December 19^th^, 2017 (fourth-year data extraction). We included the 8841 participants who completed the mood and affect module.

Approximately two years after the launch of the study, HND added a follow-up module on mood and affect to the website. Participants were invited to complete this follow-up module via an electronic newsletter. This follow-up module included the PANAS, but not the QIDS. A total of 503 participants, who filled out the PANAS twice, were included in the analyses of the second research question.

### Measures

The Positive and Negative Affect Schedule (PANAS) was originally developed by Watson and Clark to measure the relatively independent dimensions of PA and NA [34]. In their validation study of 1988 the scales were found to be internally consistent (Cronbach’s alpha 0.88 for PA and 0.85 for NA and largely uncorrelated (-0.22), sharing approximately 1% to 5% of their variance. These results were replicated in a large non-clinical sample by Crawford and Henry who found a Cronbach’s alpha of 0.89 for PA and 0.85 for NA, and a correlation between PA and NA of -.31 for males and -.24 for females [35]. Crocker, De Paoli and Sweeny, and Melvin and Molly found comparable results [36–38]. Hill et al. found an internal consistency of 0.84 for PA and 0.80 for NA in a sample of older Dutch adults (age 42–84) [39]. NA is broadly correlated with symptoms of anxiety and depression and is a general predictor of psychiatric disorder. PA is more specifically negatively related to symptoms of depression (anhedonia and depressed affect) and interpersonal anxiety (social phobia) [40–42].

We used the Flemish version of the PANAS, which assesses 10 positive and 10 negative emotions over the previous week, on a Likert scale ranging from 1 to 5 (1 = very slightly or not at all; 5 = extremely), resulting in a score of 10–50 for PA and NA [43]. In a Dutch population of healthy volunteers, Peeters et al. also found a good internal consistency for PA (alpha 0.79) and NA (alpha 0.83). Peeters et al. calculated percentile scores for PA and NA in men and women. For both sexes the norm scores for PA were: very low (≤ 26), low (27–29), below average (30–31), average (32–34), above average (35–37), high (38–41), and very high (≥ 42). For men and women respectively, the norm scores for NA were: very low (- / ≤ 12), low (≤ 12, 13–14), below average (13–15, 15–16), average (16–17, 17–20), above average (18–23, 21–24), high (24–28, 25–33), and very high (≥ 29, ≥ 34).

The 16-item Quick Inventory of Depressive Symptomatology self-report scale (QIDS-SR16), based on the 30-Item Inventory of Depressive Symptomatology (IDS), was developed by Rush [44, 45]. We used the Dutch version of the QIDS-SR16 (www.ids-qids.org), which is widely used in Dutch clinical practice and research [31, 46–48]. The IDS and QIDS were designed to measure the overall severity of DSM major depressive disorder by assessing each of the nine symptom domains defining the syndrome: sad mood, concentration, self-criticism, suicidal ideation, interest, energy/fatigue, sleep, change in appetite, weight, and psychomotor activity, all measured over the previous week [49]. The QIDS-SR16 has good psychometric properties and is sensitive to change [44, 45, 50]. According to Rush and Trivedi, the correlation between the IDS-SR30 and QIDS-SR16 lies between 0.83 and 0.96 [44, 45]. In a systematic review considering the psychometric properties of the QIDS-SR, Reilly et al. found Cronbach’s alphas ranging from 0.69 to 0.89 for the QIDS-SR [50].

The QIDS covers nine symptom domains of depression [51]. It assesses the six domains sad mood, concentration, energy, interest, guilt, suicidal ideation/plans, with one item (range 0–3). Four items of the QIDS cover the sleep domain (insomnia and hypersomnia), and two items cover the psychomotor activity domain (agitation and retardation). Four items assess the appetite and weight domain (increase or decrease). The highest rating on any one relevant item of these last three domains is used to score this domain (range 0–3). The QIDS total score ranges from 0 to 27 [52]. Cut-off scores were derived by Rush et al. through item-response theory and calibrated against a reference standard of the Hamilton Depression Rating scale indicate 0–5 no depression, 6–10 mild, 11–15 moderate, 16–20 severe, 21–27 very severe depression [44, 50].

In order to answer the first research question, not only seasonal differences in the QIDS total score were examined but also differences in eight separate items of the QIDS, which had been selected for their assumed relationship with seasonality. The Seasonal Pattern Assessment Questionnaire, a self-rating screening questionnaire that retrospectively measures seasonal variation in mood-related domains, also assesses these items [53, 54].

The 60-item NEO-Five-Factor Inventory was used to measure the neuroticism domain, which describes the tendency to experience negative emotions and psychological distress in response to stressors [55]. The inventory is composed of descriptive statements (e.g. “I am not a worrier”, “I feel inferior”) rated on a 5-point Likert-type scale (1 = strongly disagree to 5 = strongly agree). Scores are calculated by summing the 60 items and can be recalculated to norm scores, which were validated for the Dutch population [55].

The start and completion dates and times of the questionnaires were recorded automatically and were categorised into the four seasons (spring: March 21–June 20, summer: June 21–September 20, autumn: September 21–December 20, and winter: December 21–March 20). In order to avoid erroneous allocation of the assessments to the seasons we only included participants in this study who completed the PANAS or QIDS within 84 hours (3.5 days).

### Statistical analyses

In line with prior studies, the following demographic variables were selected as potential covariates: age, gender, partner status, employment status, income and education level. ANOVA was used to test whether these variables showed seasonal differences in their distribution, which turned out to be true for all of these demographic variables. These variables served as covariates in all subsequent models on differences between the seasons in the mean scores on the PANAS and the QIDS (ANCOVA). Confidence intervals were based on 1000 bootstrap samples because of the skewness of the distribution of the QIDS and negative affect scores. Post-hoc tests were used to examine the differences between the four seasons. In a subsequent model, we tested whether the variable neuroticism (continuous variable) moderated the seasonal effect by adding neuroticism and the interaction between neuroticism and season. In order to simplify the interpretation of a significant interaction, we performed a follow-up analysis. The participants were categorised as either low or high on neuroticism. We used a median split, and the model was rerun with this variable. We were not able to perform an a priori sample size calculation because the HND study was not primarily targeted at studying seasonality. Instead, we performed a post hoc power calculation.

For the second research question, repeated-measures ANOVA was used to test whether there were within-subject differences in the mean scores of the PANAS for the two different times the questionnaire was filled out, using pairs of seasons (e.g. spring-winter). This was done only for pairs of seasons with sufficient observations. The necessary sample size (N = 16) was calculated to detect a large effect (Cohens d = 0.8), with a power of 90% and a correlation between repeated measures of 0.6. A check was performed to see whether the seasonal order of the measurements made a difference. This was done to rule out the possibility that the second assessment was always lower or higher due to an unknown confounding factor. The final model tested whether neuroticism moderated the within-subject seasonal effect by adding neuroticism and the interaction between neuroticism and season. For the sake of interpretation and visualisation of the interaction effect we also created a categorical neuroticism variable, using a median split (low neuroticism ranging from 0 to 32, high neuroticism ranging from 33 to 60). SPSS version 25 was used to analyse the data [56]. A two-sided P-value below .05 was considered significant.

## Results

### Demographic characteristics

Table 1 shows the seasonal distribution of the demographic variables for the 8841 participants who filled out the mood and affect module. The mean age of the sample was 45.4 years (SD 14.5), with 68.7% of the participants being women. There was an increase in subscriptions following the publication of an article about the project in a national newspaper on April 19^th^, 2014 [57]. This is why a majority of participants completed the questionnaires in spring. The latter respondents were significantly older, more often male, in a relationship, employed, with a higher income, and had higher education levels than participants who filled out the questionnaires in the other seasons.

**Table 1:** Seasonal distribution of demographic variables.

	Spring	Summer	Autumn	Winter	Total	Overall Test^a^	p
n, (%)	5852 (66.2%)	455 (5.1%)	1206 (13.6%)	1328 (15.0%)	8841 (100%)		
Age at baseline (y), mean (SD)	47.6 (14.0)	39.3 (14.4)	42.1 (14.3)	40.6 (14.4)	45.4 (14.5)	F (3, 8837) = 148.7	< .001
Female, n (%)	3859 (65.9%)	345 (75.8%)	869 (72.1%)	1003 (75.5%)	6076 (68.7%)	X^2^ (3) = 66.6	< .001
Partner	4429 (75.7%)	315 (69.2%)	857 (71.1%)	886 (66.7%)	6487 (73.4%)	X^2^ (3) = 53.4	< .001
Employed	4429 (75.7%)	338 (74.3%)	833 (69.1%)	918 (69.1%)	6518 (73.7%)	X^2^ (3) = 39.6	< .001
Monthly income < € 1500^b^	408 (11.8%)	53 (23.1%)	165 (22.7%)	232 (25.0%)	858 (16.1%)	X^2^ (3) = 133.0	< .001
Monthly income € 1500—€ 3500^b^	1605 (46.5%)	104 (45.4%)	323 (44.5%)	430 (46.3%)	2462 (46.1%)	X^2^ (3) = 1.0	0.79
Monthly income > € 3500^b^	1438 (41.7%)	72 (31.4%)	238 (32.8%)	267 (28.7%)	2015 (37.8%)	X^2^ (3) = 66.1	< .001
Education level^c^, mean (SD)	7.05 (1.1)	6.98 (1.2)	6.76 (1.3)	6.74 (1.3)	6.96 (1.2)	F (3, 8753) = 52.6	< .001

^a^Based on Chi-square test for categorical variables and ANOVA for continuous variables

^b^n = 5,335

^c^Education level ranging from 1 to 8, n = 8,757

The questions concerning income and education were missing for 3506 and 84 participants respectively, leaving 5282 participants for further analysis of the seasonal difference in scores on the PANAS and QIDS. A post hoc power calculation on the sample of 5282 participants who were included in the cross-sectional analysis showed a power of 0.90 to detect even a small effect (Cohens D = 0.247) between the two smallest groups: 229 participants in summer and 717 participants in autumn. The power calculation confirmed that our sample size was sufficient. In the analysis with neuroticism as a moderator variable, a total of 4026 participants, those who completed the NEO-FFI-3, were included.

A total of 503 participants completed the follow-up module and filled out the PANAS twice. In Table 2, we compare the demographic variables of the participants who completed the follow-up module to those who did not. The proportion of women in the follow-up module was not statistically different from the participants who did not complete the follow-up module. The participants who filled out the PANAS twice were on average 2.3 years older than the participants who did not complete the follow-up module. They were less frequently involved in a partner relationship than participants from the original sample. The mean education level of the participants in the follow-up module was slightly higher than that of the participants in the original sample. They did not differ significantly on other demographic variables from the participants who did not complete the follow-up module. The mean interval between the first and second measurements was 22 months. The minimum interval between measurements was three months, meaning that none of the participants completed both measures within one season.

**Table 2:** Seasonal distribution of demographic variables in the subsample of respondents with repeated measures.

	Total	No Repeated measures subsample	Repeated measures subsample	Overall Test^a^	p
	n = 8841	n = 8338	n = 503		
Age at baseline (y), mean (SD)	45.4 (14.5)	45.2 (14.5)	47.5 (14.3)	F (1, 8840) = 11.1	0.001
Female, n (%)	6076 (68.7%)	5725 (68.7%)	351 (69.8%)	X^2^ (1) = 0.3	0.599
Partner	6487 (73.4%)	6148 (73.7%)	339 (67.4%)	X^2^ (1) = 9.8	0.002
Employed	6518 (73.7%)	6163 (73.9%)	355 (70.6%)	X^2^ (1) = 2.7	0.099
Income per month < € 1500^b^	858 (16.1%)	788 (16.1%)	70 (15.7%)	X^2^ (1) = 0.05	0.833
Income per month € 1500—€ 3500^b^	2462 (46.1%)	2241 (45.8%)	221 (49.7%)	X^2^ (1) = 2.4	0.120
Income per month > € 3500^b^	2015 (37.8%)	1861 (38.1%)	154 (34.6%)	X^2^ (1) = 2.1	0.151
Education level^c^, mean (SD)	6.96 (1.2)	6.95 (1.2)^d^	7.12 (1.1)^e^	F (1, 8756) = 13.8	0.002

^a^: Based on Chi-square test for categorical variables and ANOVA for continuous variables

^b^: n = 5335

^c^: Education level ranging from 1 to 8, n = 8757

^d^: n = 8256

^e^: n = 501

### Seasonal differences in mood and affect on a population level

**Fig 1:**
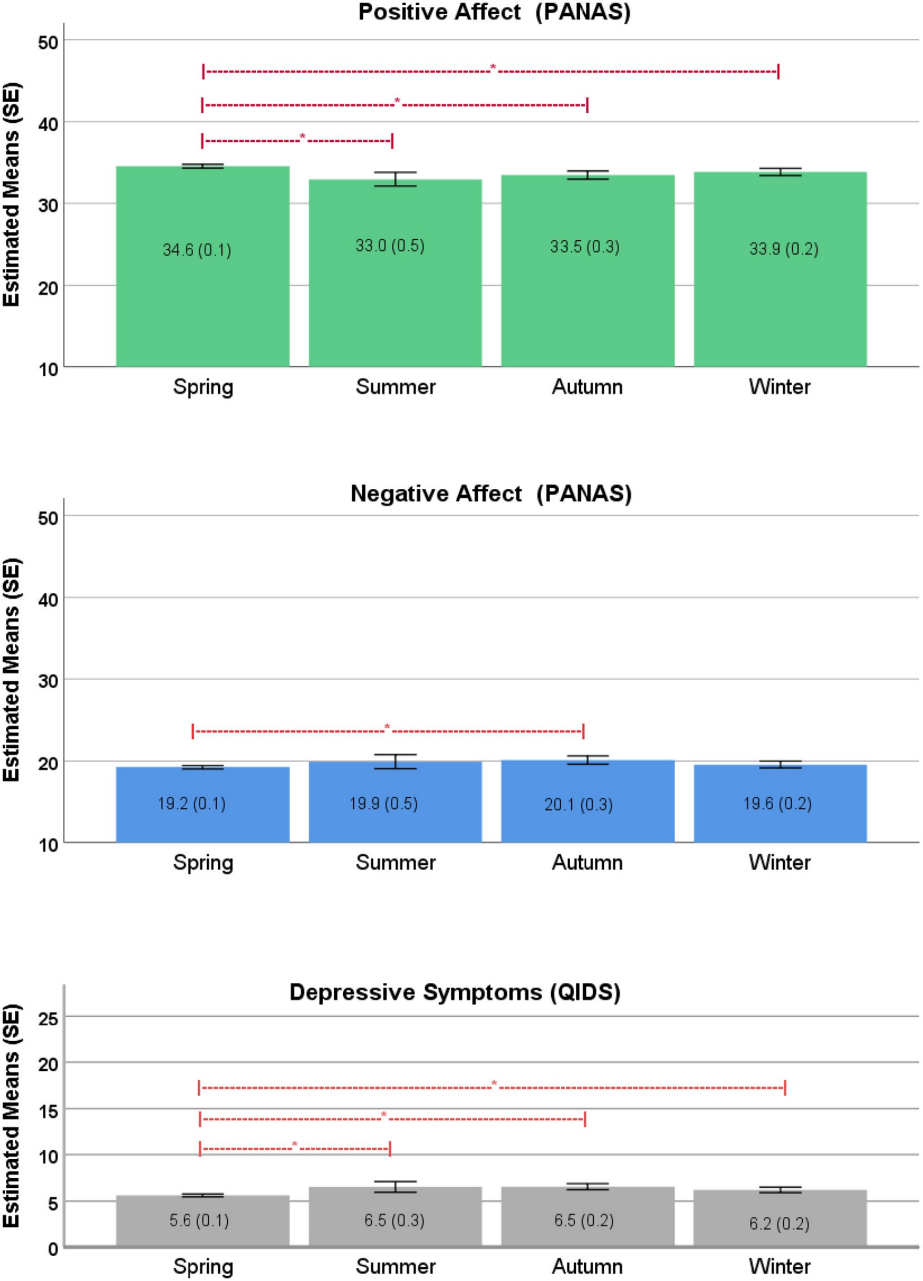
Positive and negative affect (PANAS) and depressive symptoms (QIDS) by season. Estimated means and standard errors (SE) based on 1000 bootstrap samples from ANCOVA models adjusted for age, gender, partner status, education, employment status, and income.

The Pearson correlation between the QIDS and PA was -0.69 (p < 0.01) and between the QIDS and NA 0.71 (p < 0.01). Table 3 shows the estimated mean scores per season of the PANAS and QIDS after adjustment for covariates. The overall test for the adjusted mean differences between the seasons was significant for all scales. Post-hoc tests for the PANAS and QIDS showed that participants scored significantly higher on the positive affect scale in spring when compared to summer (estimated mean difference 1.6, 95% CI 0.7 to 2.5, p = 0.001), autumn (estimated mean difference 1.1, 95% CI 0.5 to 1.6, p = 0.002), and winter (estimated mean difference 0.7, 95% CI 0.1 to 1.3, p = 0.012) (Fig 1, upper panel), and significantly lower on the negative affect scale in spring when compared to autumn (estimated mean difference -0.9, 95% CI -1.5 to -0.5 p = 0.002) (Fig 1, middle panel). Participants scored significantly lower on the QIDS in spring when compared to summer (estimated mean difference -0.9, 95% CI -1.5 to -0.3, p = 0.003), autumn (estimated mean difference -0.9, 95% CI -1.3 to -0.6, p = 0.001), and winter (estimated mean difference -0.5, 95% CI -1.0 to -0.2, p = 0.004) (Fig 1, lower panel). Small effect sizes (> = 0.20) were found for the difference in positive affect between spring and summer (0.25) and the difference in QIDS score between spring and summer (0.20), spring and autumn (0.20), spring and winter (0.13). The other effect sizes were very small (0.10–0.15).

**Table 3:** Seasonal distribution of positive affect, negative affect and depressive symptoms.

	Spring n = 3414 (64.6%)	Summer n = 229 (4.3%)	Autumn n = 717 (13.6%)	Winter n = 922 (17.5%)	Total n = 5282 (100%)	Overall F-Test	p
PANAS Positive Affect Scale, mean (SE)	34.6 (0.1)	33.0 (0.5)	33.5 (0.3)	33.9 (0.3)		F (3, 5271) = 8.9	<0.001
PANAS Negative Affect Scale, mean (SE)	19.2 (0.1)	19.9 (0.5)	20.1 (0.3)	19.6 (0.2)		F (3, 5271) = 3.8	0.010
QIDS, mean (SE)	5.6 (0.1)	6.5 (0.3)	6.5 (0.2)	6.2 (0.2)		F (3, 5271) = 12.0	<0.001

Estimated means and standard errors (SE) based on 1,000 bootstrap samples from ANCOVA models adjusted for age, gender, partner status, education, employment status, and income.

### Seasonal differences in eight separate questions of the QIDS

Table 4 shows the estimated mean scores per season on eight separate “seasonality-related” questions of the QIDS. The overall test for the adjusted mean differences between the seasons was significant for all items except “decreased weight”, “increased weight” and “increased appetite”. The post hoc test for the question “sleeping too much” showed that participants slept more in winter compared to spring. Participants scored higher for loss of general interest in autumn and winter compared to spring. Participants felt less sad in spring than in summer, autumn and winter. Decreased appetite was more prominent in summer and autumn than in spring. Participants had less energy in autumn and winter compared to spring and less energy in winter compared to autumn. The effect sizes were very small (0.10–0.19) for most of the estimated mean differences. Only the difference between spring and autumn in energy could be considered small (0.24). The estimated differences and effect sizes are shown in Table 5.

**Table 4:** Seasonal distribution of symptoms associated with winter depression.

	Spring n = 3414 (64.6%)	Summer n = 229 (4.3%)	Autumn n = 717 (13.6%)	Winter n = 922 (17.5%)	Total n = 5282 (100%)	Overall F-Test	p
Sleeping too much, mean (SE)	0.30 (0.01)	0.36 (0.04)	0.32 (0.02)	0.37 (0.02)		F (3, 5271) = 4.5	<0.003
General interest, mean (SE)	0.31 (0.01)	0.37 (0.04)	0.44 (0.03)	0.37 (0.02)		F (3, 5271) = 8.4	<0.001
Feeling sad, mean (SE)	0.62 (0.01)	0.73 (0.05)	0.74 (0.03)	0.68 (0.03)		F (3, 5271) = 7.1	<0.001
Decreased weight (within last 2 weeks)	0.18 (0.01)	0.15 (0.04)	0.16 (0.03)	0.14 (0.02)		F (3, 4727) = 1.1	0.353
Increased weight (within last 2 weeks)	0.18 (0.01)	0.21 (0.05)	0.23 (0.03)	0.23 (0.02)		F (3, 4859) = 2.5	0.061
Decreased appetite	0.08 (0.01)	0.17 (0.04)	0.15 (0.02)	0.10 (0.02)		F (3, 4791) = 7.4	<0.001
Increased appetite	0.16 (0.01)	0.19 (0.04)	0.21 (0.03)	0.20 (0.02)		F (3, 4920) = 1.5	0.209
Energy level	0.57 (0.01)	0.64 (0.05)	0.75 (0.03)	0.64 (0.03)		F (3, 5271) = 12.5	<0.001

Estimated means and standard errors (SE) based on 1,000 bootstrap samples from ANCOVA models adjusted for age, gender, partner status, education, employment status, and income.

**Table 5:** Post hoc tests; estimated mean differences between the seasons in eight separate questions of the QIDS.

Season*	QIDS question	Estimated difference	95% Confidence interval Lower bound Upper bound		P	Effect size
**Spring–Summer**	Feeling sad	-0.12	-0.23	0.00	0.028	0.15
	Decreased appetite	0.09	0.02	0.16	0.016	0.20
**Spring–Autumn**	Feeling sad	-0.13	-0.20	-0.06	0.001	0.16
	Decreased appetite	0.06	0.02	0.11	0.002	0.15
	General interest	0.13	0.07	0.18	0.001	0.18
	Energy level	-0.19	-0.25	-0.12	0.001	0.24
**Spring–Winter**	Feeling sad	-0.07	-0.13	0.01	0,018	0.09
	General interest	0.06	0.01	0.12	0.019	0.10
	Sleeping too much	0.07	0.03	0.12	0.002	0.13
	Energy level	-0.08	-0.14	-0.02	0.013	0.10
**Autumn–Winter**	Energy level	-0.11	-0.19	-0.02	0.012	0.13

* Only significant differences have been included.

### Moderation by neuroticism of seasonality in mood and affect

**Fig 2:**
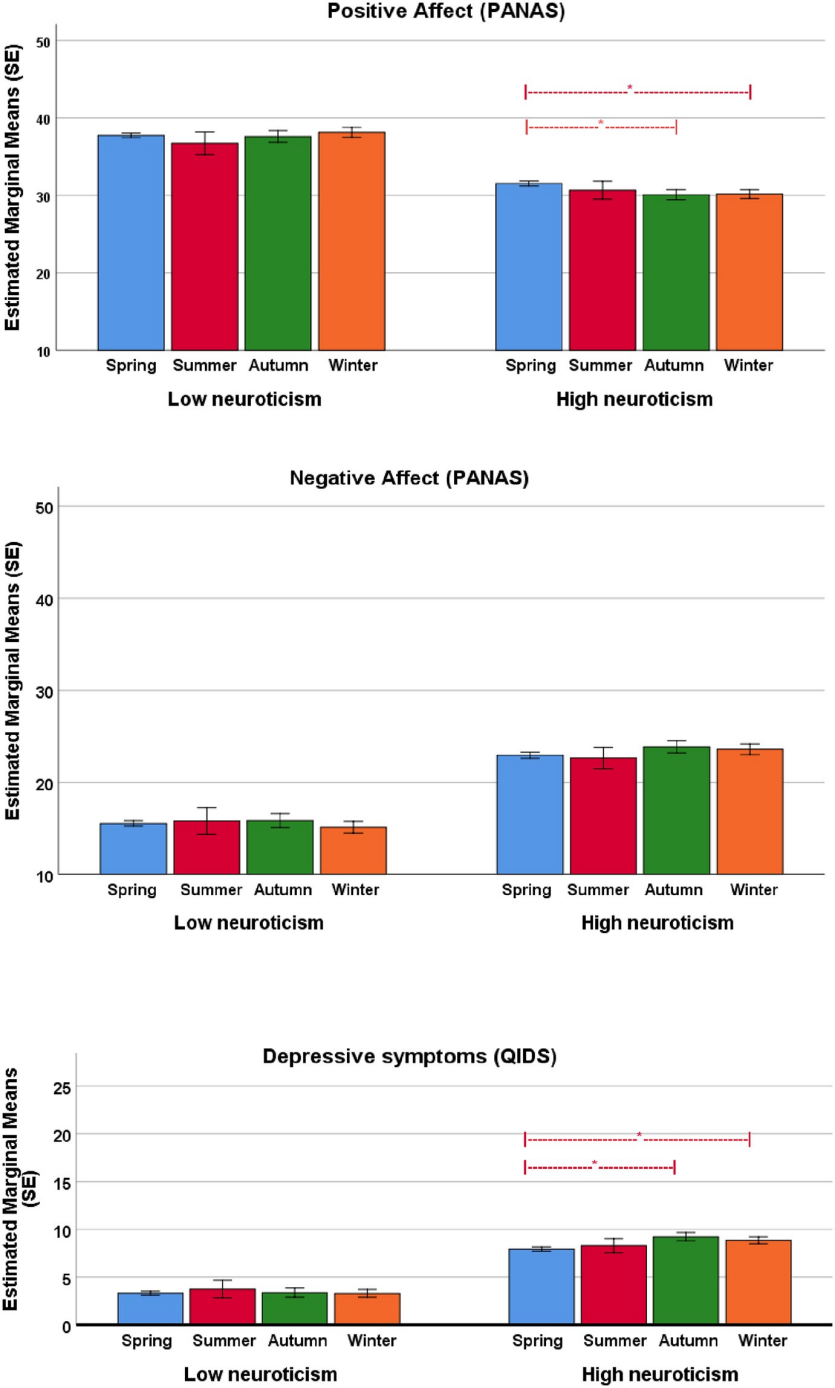
Positive and negative affect (PANAS) and depressive symptoms (QIDS) by season for high and low neurotic respondents. Estimated means and standard errors (SE) based on 1,000 bootstrap samples from ANCOVA models adjusted for age, gender, partner status, education, employment status, and income.

Subsequent analyses with the continuous variable neuroticism showed that this personality factor significantly moderated the effect of season in the model for PA: F (3, 4023) = 4.69, p = 0.003, NA: F (3, 4023) = 3.1, p = 0.027, and the QIDS: F (3, 4023) = 10.7, p < 0.001. Elaboration of this interaction effect for the PA model showed that seasonal effects were present only in high-neuroticism participants (Fig 2, upper panel): in this subgroup, PA scores were higher in spring compared to autumn and winter. In the NA model, no significant differences between the seasons were observed for either the low or high neurotic group (Fig 2, middle panel). Only a trend of lower NA scores in spring in the high-neuroticism subgroup was observed (p = 0.051). For the depressive symptoms (QIDS) model, seasonal effects were also present only in high-neuroticism participants (Fig 2, lower panel): QIDS scores were lower in spring compared to autumn and winter. The effect sizes were very small or small (0.19–0.25).

### Seasonality in repeated measures of mood and affect

For the analysis of the within-subject seasonality effects based on the repeated measures of the PANAS, the following pairs of seasons were included on the basis of sufficient observations: spring–autumn, spring–winter, summer–winter, autumn–winter. Table 6 shows the estimated mean differences in scores on the PANAS for the repeated measures in these pairs of seasons. The overall test for the mean differences between the seasons was not significant for any of the scales. A trend could be observed for PA: participants scored higher in spring compared to winter (p = 0.050).

**Table 6:** Mean difference in positive and negative affect between the seasons in repeated measures.

PANAS		Positive Affect			Negative Affect		
Pairs of Seasons	N	Mean Difference (SE)	F Test	P	Mean Difference (SE)	F Test	P
Spring—Autumn	34	0.3 (1.2)	F (1, 33) = 0.077	0.783	0.03 (1.1)	F (1, 33) = 0.001	0.979
Spring—Winter	218	0.8 (0.4)	F (1, 217) = 3.898	0.050	-0.3 (0.4)	F (1, 217) = 0.471	0.493
Summer—Winter	16	-0.4 (1.8)	F (1, 15) = 0.061	0.809	-2.8 (2.1)	F (1, 15) = 1.767	0.204
Autumn—Winter	41	0.5 (0.8)	F (1, 40) = 0.306	0.583	0.3 (1.0)	F (1, 40) = 0.080	0.778
Total	503						

Mean differences based on estimated marginal means.

Re-analysis with the order of the measurement as interaction term revealed that only for the pair autumn-winter and only for the outcome PA the order of the measurement was a significant moderator of the effect (p = 0.021). Elaboration of this interaction effect by splitting the group in autumn–winter and winter–autumn showed that for these groups, there were no significant within-subject differences between the seasons for PA. As a check of whether scores were generally higher or lower when the questionnaire was filled out for a second time, we examined the scores for participants who filled out the PANAS in different years but in the same season: spring–spring (n = 118) and winter–winter (n = 63).

No mean difference between the seasons was found for any of these pairs. These checks suggest that the difference between the first and second assessments was not due to an unknown confounding factor or a test-retest effect.

The numbers of the pairs summer–summer (n = 1) and autumn–autumn (n = 3) were too small to analyse.

### Moderation by neuroticism of seasonality in repeated measures

There was no significant interaction between the seasons and neuroticism for any of the paired measurements listed in Table 6. So, neuroticism did not significantly moderate the within-subject differences between the seasons. We checked whether there was a difference in neuroticism between the participants who filled out the PANAS once (n = 4,334, mean score 32.9, SD 9.4) or twice (n = 473, mean score 33.0, SD 9.6). This was not the case (F(1, 4,805) = 0.1, p = 0.75).

## Discussion

The purpose of this study was to investigate whether mood and affect are related to the seasons. Secondly, we examined the role of neuroticism as a potential moderator of seasonality. The main findings of this study were: on a population level, participants scored higher on positive affect in spring compared to the other seasons, lower on negative affect in spring compared to autumn, and lower on QIDS depressive symptoms in spring compared to the other seasons. The same pattern was visible in the separate “seasonality-related” questions of the QIDS (except for weight change and increased appetite): participants felt less sad, slept less, had more energy, more general interest in spring compared to the other seasons, mainly autumn and winter. In summary, this study shows that participants, in general, feel better in spring compared to the other seasons, but effect sizes were small or very small. The personality factor neuroticism moderated the effect of the season in all three outcomes. There were no within-subject seasonal differences in the scores of positive and negative affect, as shown in the repeated measures analysis in participants who filled out the questionnaires twice. The power of these analyses may have been insufficient to detect significant seasonal differences, due to smaller numbers and the fact that effect sizes were already very small or small in the first group. This may also explain that neuroticism did not moderate within-subject seasonal differences.

The finding that seasonal differences were only seen in the group of high-neurotic participants is in line with our previous study, in which we hypothesised that subjects who score high on neuroticism tend to attribute their symptoms and unhappiness to the seasons [26]. This finding is also in line with the findings of Rosellini and Nooteboom that the symptoms of depression are related to the personality trait neuroticism [58, 59].

In the crowdsourcing study HND procedure, the general public volunteered to assist in scientific research. In return, participants received feedback on their scores and were able to follow the results of the research on the internet [31]. Brabham described the internet crowdsourcing procedure as a relatively new model for application in public health [60]. Possible advantages mentioned by Bevelander are that by this sampling methodology already existing hypotheses can be reproduced but also that this methodology can generate ideas that are less well-documented or otherwise tend to be overlooked [61]. In previous crowdsourcing studies, the participants recruited were more diverse than in other means of recruitment [62]. Possible disadvantages of this method are selection bias and the impossibility to calculate non-response percentages, as it is not possible to know how many people have heard of the project or visited the website but did not enter the study [63, 64]. In order to find a group of participants for HND that could be representative for the general population (and thereby attempting to reduce the limitation of selection bias), publicity for HND was sought using several newspaper articles, magazine articles, public lectures, radio interviews, and other media. In order to examine possible selection effects, Van der Krieke et al. [31] compared the HND sample with the governmental data of the general Dutch population (Central Bureau of Statistics) and two large population studies: the Netherlands Mental Health Survey (NEMESIS-2) and the Lifelines population study [32,33]. They confirmed a certain selection bias. Compared to the general Dutch population, the HND participants were more often women (65.2% versus 50.5%; NEMESIS = 55.2%, Lifelines = 57,9%), on average 6 years older (45 versus 39 years; NEMESIS = 44, Lifelines = 42), more often with a partner (74% versus 58%;), more often living together (61 versus 47%; NEMESIS = 68%) and had higher education levels (> 20 years 76% versus 35%; NEMESIS = 35%) [31].

This selection bias clearly is a limitation of the present research. Moreover, in our study, a majority of the participants completed the questionnaires in spring. Although we adjusted for the differences between the seasons due to this selective inclusion by using the demographic variables as covariates, we cannot rule out the possibility that the results were still partly due to some unmeasured confounder. Since our sample was a general population sample, another potential limitation is that the proportion of participants suffering from SAD can be expected to be low (ranging from 3%–10%), implying that the contribution of SAD patients to our study results will be limited[11].

Depressive disorders and anxiety disorders show a high comorbidity [65, 66]. For this reason, it would have been interesting to include a measure of anxiety. However, in a previous article, we showed that the administered depression scale (QIDS) and the BAI (Becks Anxiety Inventory) showed a correlation of 0.80 [27]. In the HND study, the Anxiety subscale of the Depression Anxiety Stress Scale (DASS) was used to assess anxiety over the previous week. In our data, there was a correlation of 0.70 between the QIDS and the anxiety scale of the DASS (S1 Table). Since our main objective was to investigate the seasonality of depression and positive and negative affect, we did not include this measure of anxiety as a confounder because it could have masked the seasonal effect on depression.

A strength of this study is its large sample size for the analyses in the entire group and the spring–winter group in the repeated measures analyses. Other strengths are the use of validated instruments, comparability with other Dutch population studies, the use of questionnaires covering a short period guaranteeing a relative absence of memory bias, and the inclusion of a personality factor in the analyses.

The mechanism of cognitive attribution may underlie the relation between (perceived) seasonality and neuroticism [27, 67, 68]. For future studies on seasonality of mood and behaviour, we recommend including the personality measure neuroticism and a measure to establish the attribution style. Other confounding factors like presence or absence of pre-existing physical or mental health conditions, treatment and stressful life events should be measured as well. The objective then is to further disentangle the relationship between neuroticism, attribution style and (perceived) seasonality of mood and behaviour.

## Conclusion

This general population study did not show substantial seasonal differences in mood and affect. The seasonal differences that were found had small effect sizes, and were only seen in high-neurotic participants. The seasons were related to PA in a similar way as to NA and depressive symptoms. Spring seems to be the most favourable season, with participants reporting higher PA and lower NA compared to the other seasons. The findings of this study do not support the widespread belief that seasons influence mood to a great extent, at least not in a general population sample participating in an online crowdsourcing study.

## Supporting information

S1 Table Correlations between PANAS, QIDS and DASS. Correlation is significant at the 0.01 level (2-tailed)**.
(DOCX)
