# Supplementary material for: Seasonality of mood and affect in a large general population sample
Source: PLoS One. 2020 Sep 14;15(9):e0239033. doi: 10.1371/journal.pone.0239033 (PMC7489524; doi:10.1371/journal.pone.0239033)
Supplement: S1 Table — Correlation is significant at the 0.01 level (2-tailed)**. (DOCX) [file pone.0239033.s001.docx]

|  | **PANAS PA** | **PANAS NA** | **QIDS** | **DASS Depression** | **DASS Anxiety** | **DASS**  **Stress** |
| --- | --- | --- | --- | --- | --- | --- |
| **PANAS PA** |  |  |  |  |  |  |
| **PANAS NA** | -.535** |  |  |  |  |  |
| **QIDS** | -.681** | .712** |  |  |  |  |
| **DASS Depression** | -.712** | .703** | .825** |  |  |  |
| **DASS Anxiety** | -.463** | .697** | .669** | .667** |  |  |
| **DASS**  **Stress** | -.531** | .775** | .719** | .720** | .721** |  |
